# Focusing HIV-1 Gag T cell responses to highly conserved regions by DNA vaccination in HVTN 119

**DOI:** 10.1172/jci.insight.180819

**Published:** 2024-08-01

**Authors:** Spyros A. Kalams, Barbara K. Felber, James I. Mullins, Hyman M. Scott, Mary A. Allen, Stephen C. De Rosa, Jack Heptinstall, Georgia D. Tomaras, Jiani Hu, Allan C. DeCamp, Margherita Rosati, Jenifer Bear, Michael N. Pensiero, John Eldridge, Michael A. Egan, Drew Hannaman, M. Juliana McElrath, George N. Pavlakis, HIV Vaccine Trials Network 119(HVTN 119) Study Team

**Affiliations:** 1 Division of Infectious Diseases, Department of Medicine, Vanderbilt University Medical Center, Nashville, Tennessee, USA.; 2 Department of Pathology, Microbiology and Immunology, Vanderbilt University, Nashville, Tennessee, USA.; 3 Human Retrovirus Pathogenesis Section, Vaccine Branch, Center for Cancer Research, National Cancer Institute at Frederick, Frederick, Maryland, USA.; 4 Departments of Microbiology, Medicine and Global Health, University of Washington, Seattle, Washington, USA.; 5 San Francisco Department of Public Health, San Francisco, California, USA.; 6 Department of Medicine, UCSF, San Francisco, California, USA.; 7 Division of AIDS, National Institute of Allergy and Infectious Diseases (NIAID), NIH, Rockville, Maryland, USA.; 8 Vaccine and Infectious Disease Division, Fred Hutchinson Cancer Center, Seattle, Washington, USA.; 9 Duke Center for Human Systems Immunology, Departments of Surgery, Integrative Immunobiology, Molecular Genetics, and Microbiology, Durham, North Carolina, USA.; 10 Auro Vaccines LLC (formerly Profectus BioSciences, Inc.), Pearl River, New York, USA.; 11 Ichor Medical Systems, San Diego, California, USA.; 12 Human Retrovirus Section, Vaccine Branch, Center for Cancer Research, National Cancer Institute at Frederick, Frederick, Maryland, USA.; 13 The HVTN 119 Study Team is detailed in Supplemental Acknowledgments.

**Keywords:** AIDS/HIV, Clinical trials, AIDS vaccine

## Abstract

**BACKGROUND:**

An HIV-1 DNA vaccine composed of 7 highly conserved, structurally important elements (conserved elements, CE) of p24^Gag^ was tested in a phase I randomized, double-blind clinical trial (HVTN 119, NCT03181789) in people without HIV. DNA vaccination of CE prime/CE+p55^Gag^ boost was compared with p55^Gag^.

**METHODS:**

Two groups (*n* = 25) received 4 DNA vaccinations (CE/CE+p55^Gag^ or p55^Gag^) by intramuscular injection/electroporation, including IL-12 DNA adjuvant. The placebo group (*n* = 6) received saline. Participants were followed for safety and tolerability. Immunogenicity was assessed for T cell and antibody responses.

**RESULTS:**

Both regimens were safe and generally well tolerated. The p24CE vaccine was immunogenic and significantly boosted by CE+p55^Gag^ (64% CD4^+^, *P* = 0.037; 42% CD8^+^, *P* = 0.004). CE+p55^Gag^ induced responses to 5 of 7 CE, compared with only 2 CE by p55^Gag^ DNA, with a higher response to CE5 in 30% of individuals (*P* = 0.006). CE+p55^Gag^ induced significantly higher CD4^+^ CE T cell breadth (0.68 vs. 0.22 CE; *P* = 0.029) and a strong trend for overall T cell breadth (1.14 vs. 0.52 CE; *P* = 0.051). Both groups developed high cellular and humoral responses. p24CE vaccine–induced CD4^+^ CE T cell responses correlated (*P* = 0.007) with p24^Gag^ antibody responses.

**CONCLUSION:**

The CE/CE+p55^Gag^ DNA vaccine induced T cell responses to conserved regions in p24^Gag^, increasing breadth and epitope recognition throughout p55^Gag^ compared with p55^Gag^ DNA. Vaccines focusing immune responses by priming responses to highly conserved regions could be part of a comprehensive HIV vaccine strategy.

**TRIAL REGISTRATION:**

Clinical Trials.gov NCT03181789

**FUNDING:**

HVTN, NIAID/NIH

## Abstract 2

A novel vaccine is shown to induce broad T-cell immune responses to highly conserved regions of HIV Gag, associated with long-term control of viremia.

## Introduction

Protection against HIV acquisition by vaccination is challenging, partly because the virus mutates rapidly, resulting in viable adaptations within epitope sequences targeted by the host immune system. Many strategies have been tested for the viral variability problem. One approach used herein is to target the conserved elements (CE) of the HIV-1 proteome. Cytotoxic T lymphocyte (CTL) responses to Gag, particularly its proteolytic processing product p24^Gag^, have been associated with greater HIV viremia control (1–10). CD4^+^ T cell responses to HIV negatively correlated with degree of viremia (11) and strongly associated with magnitude of CTL responses (12) and with generation of neutralizing antibody responses (13). In macaques, prechallenge Gag-specific T cell responses associated with reduced acute viremia after DNA vaccination (14–16). HIV’s ability to adapt to both CD4^+^ (17) and CD8^+^ T cell responses (18–20) suggests targeting conserved regions within HIV-1 Gag, which cannot easily escape by mutation, could be part of a comprehensive vaccine strategy.

Vaccine designs are developed to maximize coverage of HIV T cell targets (reviewed in ref. 21; references therein), as CTL presence plays an important role as a secondary defense and may synergize with antibody to block virus at the port of entry (22). Our strategy for the design of CE vaccines (2, 23, 24) was developed based on the following (3, 6, 9, 25–36): (i) identifying and mapping T cell epitopes associated with virus control (focusing on the Gag protein) in large cohorts who are long-term controllers, then including these epitopes and (ii) sequences that are stringently conserved, irrespective of associations with control. The latter were included because virologic control is associated with epitopes that are subdominant in their recognition and may not be demonstrably recognized in most HIV acquisitions. Finally, (iii) the vaccine excluded most variable epitopes, which, as often immunodominant, may act as immunologic decoys by effectively competing for immune recognition over epitopes associated with control (37–41). The only variable epitopes included were those that, if mutated, were associated with diminished viral load and long-term survival. The final constructs included sequences that were enriched in those that, if mutated, would destroy viral infectivity or impair viral fitness in cell culture (42) and were expected to be recognized in the context of broad HLA allele coverage, independent of the beneficial HLA class I genotypes B27 and B57. The 7 identified CE represent 54% of the HIV-1 p24^Gag^ protein and are present in nearly every HIV-1 (M group) strain observed to date throughout the world. A structural basis for p24^Gag^ CE conservation was supplied by the demonstration that they are enriched in viral capsid hexamer contact residues and thus critical to viral capsid integrity (42, 43). These CE were clearly subdominant in animal models, as vaccination with DNA expressing the full-length p55^Gag^ protein elicited poor or no immune responses recognizing them, though responses to epitopes outside of the CE were readily induced in mice and macaques (44, 45).

p24CE1/2 DNA, also referred to as p24CE DNA, encodes 2 forms of the 7 CE sequences (p24CE1 and p24CE2), with each CE differing by a single amino acid. This formulation captures the breadth of known variation of HIV. Despite their poor immunogenicity in the context of the full-length p55^Gag^ protein (44, 45), when expressed from the p24CE DNA vaccine, such regions were immunogenic and efficiently primed immune responses in mice and macaques (45). CE responses were also found to be boosted by subsequent vaccination with DNA expressing the full-length p55^Gag^ protein or a combination of DNA expressing CE+p55^Gag^ (45, 46). This prime-boost regimen induced immune responses with superior magnitude and breadth compared with the conventional p55^Gag^ DNA vaccine alone. It provides an effective strategy to broaden responses against highly diverse pathogens like HIV by focusing responses to critical viral elements for which few if any escape pathways exist (44, 45). The p24CE DNA prime followed by CE+p55^Gag^ DNA boost regimen induced higher and broader immune responses relative to each vaccine component alone in macaques (45, 46).

HVTN 119 was a phase I clinical trial to evaluate the safety and immunogenicity of DNA vaccines expressing HIV M Group p24^Gag^ CE and/or p55^Gag^, by intramuscular electroporation (IM EP), in adults without HIV. The vaccines were coadministered with IL-12 DNA because its inclusion has shown immunological benefit in several preclinical (47, 48) and clinical trials (49–54). A secondary objective was to rank the levels of the immune responses to CE compared with vaccination with p55^Gag^ DNA.

## Results

### Study population characteristics.

Participants were healthy adults without HIV, 18 to 50 years of age, enrolled at clinical research sites in Atlanta, Georgia; Cleveland, Ohio; and San Francisco, California. Fifty-six people participated: 23 were assigned male sex at birth, and 33 were assigned female sex at birth. Participants identified as White (59%), African American (18%), other race (9%), multiracial (7%), Asian (4%), or Native American/Alaska Native (2%) (Table 1).

### Study.

The primary objective was to evaluate the safety and tolerability, and secondarily the immunogenicity, of the HIV-1 p24CE and p55^Gag^ DNA vaccines administered by IM injection with EP in combination with IL-12 DNA. The trial was composed of 3 arms (Table 2). A heterologous prime-boost immunization protocol (Table 3) that included a p24CE DNA prime (CE), followed by a p24CE+p55^Gag^ DNA (CE/CE+gag) as boost (test group 1, T1), was compared with an arm in which all immunizations were full-length p55^Gag^ DNA (gag/gag; test group 2, T2) and a third arm consisting of placebo controls. Four immunizations were administered, with the prime formulation given at months 0 and 1 and the boosters at months 3 and 6. Cellular and humoral immune responses in vaccine recipients after the second and fourth immunizations were compared with responses in a control group given placebo injections (referred to as the control arm).

The protocol began enrolling in November 2017, and follow-up was completed in October 2019 (Table 2). Fifty participants received the active study products, and 6 received the placebo. Overall, 17 in T1 and 15 in T2 received study product at each of their 4 vaccination visits, with the remainder receiving study product at between 1 and 3 vaccination visits. Missed injections were distributed among study groups as follows: 3 of 6 participants in the placebo group, 8 of 25 participants in T1, and 10 of 25 participants in T2. The overall retention in the study was good, with 88% of the participants remaining in the study through their final clinic visit and 80% participating in their final health contact.

### Pain scores and reactogenicity.

Before and after vaccination, participants were assessed for systemic symptoms of increased body temperature, malaise and/or fatigue, myalgia, headache, chills, arthralgia, nausea, and vomiting; local symptoms of pain and/or tenderness and erythema and/or induration proximal to the injection site; and enlargement or tenderness of proximally draining lymph nodes. Pain related to EP was assessed using the Visual Analog Scale (VAS), and acceptability was assessed using a questionnaire. Social impacts of study participation were also assessed.

Episodes of deltoid pain and/or tenderness and erythema and/or induration were no greater than moderate severity at all time points, though 4 participants declined further vaccinations because of pain. Systemic reactogenicity symptoms were most frequently mild, with only 2 participants reporting severe symptoms. Fever was reported in 2 participants and was mild.

### Adverse events.

Device-related adverse events (AEs) including postinjection lightheadedness related to the EP procedure (4 episodes in 3 participants, categorized as mild), erythema at the site of injection in 1 participant (mild), and syncope with syncope-related seizure activity after the first injection in 1 participant (severe). The latter person recovered quickly but did not receive any further vaccinations. The event was reviewed by an expert committee and was considered related to the injection procedure. The most frequently reported AE (*n* = 10, 17.9%) was upper respiratory tract infections, all of which were assessed as unrelated. No deaths, cases of HIV acquisitions, or pregnancies were reported.

### EP device experience.

Among the first approximately 300 injections, difficulties in having the EP device removed from the arm occurred in 5 different participants. In 2 cases, the cause of difficulty was a bent electrode, but in the other 3 cases, it remains unknown why there was resistance to removal.

Eighteen participants (including the 5 described above) did not receive bilateral EP at 1 or more vaccination time points because of issues with use of the EP device, including device error codes related to detection of improper electrode insertion via the electrical resistance check performed before study agent injection. Specifically, this included detection of resistance values consistent with bent electrodes and/or resistance values were inconsistent with insertion into skeletal muscle (e.g., high resistance levels associated with subcutaneous tissue). Eight of these 18 participants received only 1 of the bilateral study agent injections at 1 or more time points (“half-dose”). This is because study product was administered before EP, leading to instances where study product was administered, but EP could not be performed at the injection site. The majority of these events were identified early in the study and appeared to be linked to problems with the electrode array, although no specific root cause for the errors was determined. A parallel study in people living with HIV (PLWH), using an updated model of the same EP device and a similar vaccination schema, was performed without these problems (55). Missed EPs after study product administration were equally distributed among vaccination groups, with 2 individuals in the placebo group, 6 in T1, and 7 in T2 missing at least 1 EP at 1 or more study visits. However, immunogenicity studies were performed on all collected samples regardless of the number of vaccinations received according to our intent-to-treat protocol.

Overall, 10 participants discontinued vaccinations at some point during the study. In addition to the 4 participants who discontinued because of excessive device-related pain mentioned above, 3 discontinued because of the described device malfunctions and 3 for nondevice or non–study product reasons (1 relocation, 1 personal reason, and 1 AE of gastroenteritis occurring soon after vaccination that was assessed as unrelated to study product by the protocol team).

### DNA vaccine.

**Figure 1:**
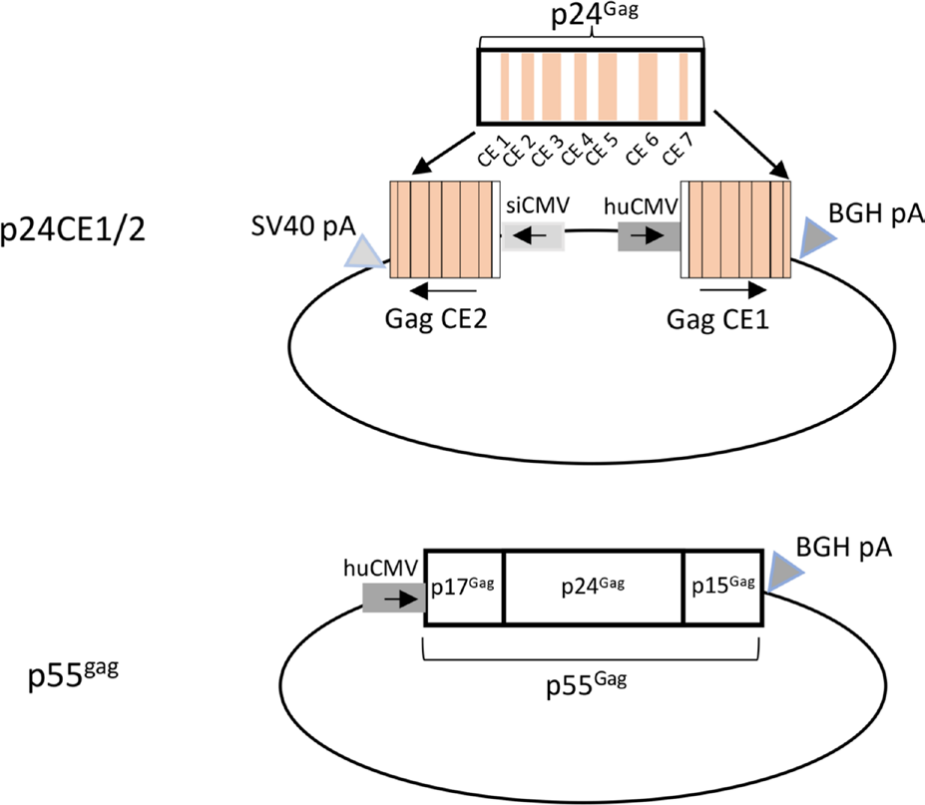
HVTN 119 DNA vaccine components. The dual-promoter CE1/2 plasmid (plasmid code 306H) expresses the 2 CE variant proteins, CE1 and CE2, using the human CMV (huCMV) and the simian CMV (siCMV) promoters, respectively. CE cassette-coding sequences are followed by the bovine growth hormone polyadenylation (BGH polyA) and the simian vacuolating virus 40 polyadenylation (SV40 polyA) signals, respectively. The 7 CE regions (CE 1 to CE 7) encompass 54% of p24^Gag^ (beige-shaded regions). The p55^Gag^ plasmid (plasmid code 114H) expresses the full-length HIV-1 HXB2 Gag from the huCMV promoter followed by the BGH polyA signal. The HIV p55^Gag^ comprises the N-terminal p17^Gag^ and p24^Gag^ and the C-terminal p15^Gag^ proteolytic cleavage products. All plasmids contain the kanamycin resistance gene in the vector backbone.

The p24CE DNA vector expressed 2 different but highly related synthetic proteins (p24CE1 and p24CE2) (44, 46) from a single plasmid encoding 2 expression-optimized p24CE genes (Figure 1). Each protein contained 7 CE regions (CE 1 to CE 7) from the p24^Gag^ protein, a proteolytic processing product of the HIV-1 p55^Gag^ precursor protein responsible for forming the viral capsid. The sequences of the p24CE1 and p24CE2 proteins are 13, 18, 24, 20, 20, 14, and 18 amino acids (AA) in length, respectively, and differ by 1 AA per CE (44) (Supplemental Table 1; supplemental material available online with this article; https://doi.org/10.1172/jci.insight.180819DS1). The length of p24CE proteins represented was in each case 124 AA, corresponding to 54% of the p24^Gag^ protein, with spacer sequences separating each CE spanning a total of 140 AA. The p55^Gag^ DNA encodes the full-length HIV-1 HXB2 p55^Gag^ protein, which encodes the proteolytic processing products p17^Gag^, p24^Gag^, and p15^Gag^ (Figure 1). IL-12 DNA (GENEVAX IL-12 plasmid) was used as an adjuvant in both arms and encodes the human IL-12 cytokine. DNA vaccines were administered by IM injection followed by in vivo EP (49, 53, 56–58). Participants received 4 vaccinations (Table 3) over 6 months administered at months (M) 0, 1, 3, and 6.

### Immunogenicity of the DNA vaccines.

**Figure 2:**
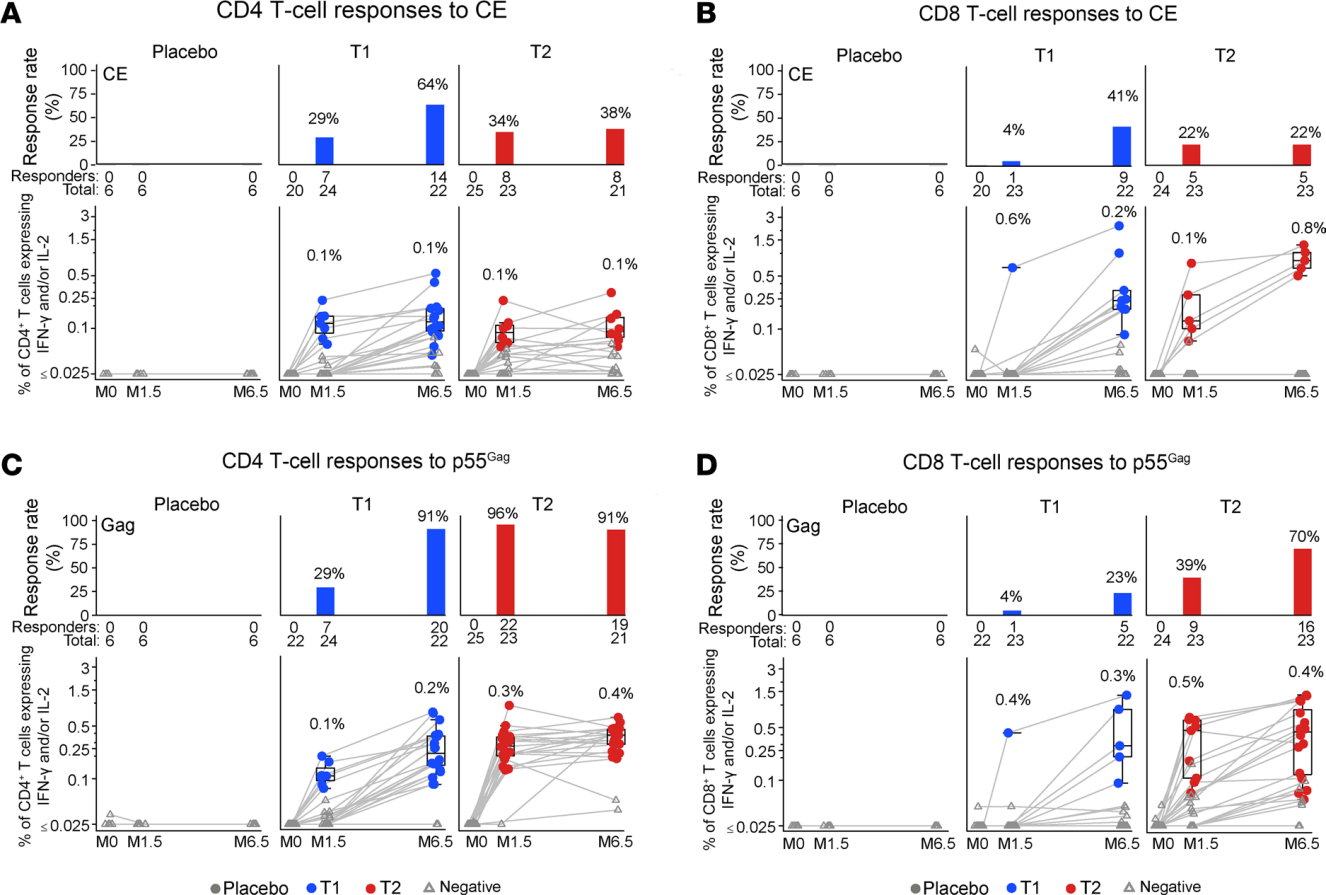
Flow cytometric analysis of cellular immune responses to CE and full-length Gag. Intracellular cytokine staining (ICS) was performed at M0, and 2 weeks after the second (M1.5) and fourth (M6.5) vaccinations, measuring antigen-specific T cell response rates (bar graphs in upper panels) and magnitudes (box-line plots in lower panels) in PBMCs against CE (**A** and **B**) and p55Gag (**C** and **D**). Response rates and the percentage of CD4^+^ and CD8^+^ T cells expressing cytokines were plotted by T cell subset, HIV peptide pool, visit, and treatment group. Samples scoring positive by Fisher’s test (see Methods) were plotted in color (coded for group) and the cases reported negative in gray. The distribution of the background-adjusted magnitude of IFN-γ^+^ and/or IL-2^+^ T cell response is displayed graphically on the log scale *y* axis, which is truncated at 0.025%; any values below this level were censored. Data points for each participant are connected by a gray line. The whiskers extend to the most extreme data points that are no more than 1.5 times the interquartile range (i.e., height of the box) or, if no value meets this criterion, to the data extremes. The CE peptide pool comprised 15-mer peptides overlapping by 11 AA and 10-mer peptides overlapping by 9 AA spanning all 7 CE. The Gag (HXB2 strain) peptide pool comprised 15-mer peptides overlapping by 11 AA spanning full-length p55^Gag^.

Three cohorts were included: T1, receiving CE/CE+ p55^Gag^ DNA; T2, receiving p55^Gag^/p55^Gag^ DNA; as well as the control group. CE- and p55^Gag^-specific T cell responses were measured at M0, 1.5, and 6.5, 2 weeks after the second and fourth vaccinations (Figure 2). All lymphocyte samples were assayed. The reasons for missing data are listed and include missed visit, visit outside of the window, participant terminated from the study, and PBMCs not collected at the time point (Supplemental Table 2). In some cases, only CD4 or CD8 data are missing based on Standard Operating Procedure–defined criteria: CD4^+^ or CD8^+^ T cells collected < 10,000, high background in the negative control (% of CD4^+^ or CD8^+^ T cells expressing IFN-γ and/or IL-2 > 0.1%), or data determined to be unreliable. Although low viability is an Standard Operating Procedure–defined criterion for not performing the assay, no data were excluded because of low viability.

No CD4^+^ or CD8^+^ T cell–positive responses were observed against CE or p55^Gag^ measured by IFN-γ and/or IL-2 (Figure 2) or by CD40L (Supplemental Figure 1) expression at M0 in the T1 and T2 groups, and there were no positive responses in the control group at any of the time points.

The first 2 vaccinations of T1 (M0, M1) with p24CE DNA were designed to focus immune responses to CE, and the booster vaccinations with both CE+p55^Gag^ DNAs at M4 and M6 were designed to increase response magnitude and breadth. In contrast, T2 received all 4 vaccinations with DNA expressing the full-length p55^Gag^.

Responses to the p24CE DNA vaccine in T1 were detected after 2 vaccinations (M1.5), with response rates of 33% based on CD40L cells (Supplemental Figure 1) and 29% based on IFN-γ or IL-2 in CD4^+^ T cells and 4% based on IFN-γ or IL-2 cells in CD8^+^ T cells (found in 1 participant) (Figure 2, A and B). Thus, the p24CE DNA vaccine is immunogenic in adults without HIV-1. The p55^Gag^ DNA vaccine in T2 also induced a CE-specific CD4^+^ T cell response with a similar rate (34% for IFN-γ or IL-2) and a 22% rate for CE-specific CD8^+^ T cells (Figure 2, A and B). At M1.5, T2 had a significantly higher proportion of p55^Gag^-specific IFN-γ^+^ or IL-2^+^CD4^+^ T cell response rate (22/23, 96%, in T2 versus 7/24, 29%, in T1; Barnard’s test *P* < 0.001) (Figure 2, C and D), as expected, since the CE proteins comprised only 25% of the full-length p55^Gag^ protein.

The response magnitude at M1.5 showed similar median levels of CE-specific CD4^+^ T cell responses in T1 and T2 (Figure 2A). As expected for the larger full-length p55^Gag^ immunogen, T2 had a higher median CD4^+^ T cell response magnitude (0.3%) to p55^Gag^ compared with T1 (0.1%) (Barnard’s test *P* < 0.001) (Figure 2C). The CD8^+^ T cell response magnitudes were overall low to CE peptide pool (Figure 2B), and as expected, responses to p55^Gag^ were higher in T2 (*P* = 0.002, Barnard’s test) (Figure 2D).

Vaccinations 3 and 4 (M4, M6) were designed primarily to boost CE-specific responses in T1. Of note, only a half dose of each plasmid (2 mg each) was used in T1 versus 4 mg in T2, which was necessary to maintain the total amount of DNA administered. At M6.5 (2 weeks following the fourth vaccination), the frequency of responders to total CE significantly increased in T1 from 29% to 64% (*P* = 0.037; Mann-Whitney 2-tailed *t* test) for CD4^+^ T cells but did not change in T2 (34% to 38%). Despite increased frequency of responders in T1, the overall response rate at M6.5 did not show a significant difference from T2 (*P* = 0.1) (Figure 2A and Supplemental Figure 1). The CD8^+^ T cell responses to CE peptides followed a similar trend, with a significant increase in the frequency of responders in T1 from 4% to 41% (*P* = 0.004; Mann-Whitney 2-tailed *t* test) and no change (*P* = 0.2) in frequency in T2 across both time points at 22% (Figure 2B) at M6.5.

As expected from the inclusion of DNA expressing full-length p55^Gag^ in the booster vaccinations in T1, there was a large increase of Gag-specific CD4^+^ T cells and the frequency of responders (to 91%) at M6.5 (Figure 2C and Supplemental Figure 1), while the response rate in T2 remained high at 91%. The CD8^+^ T cell response rate increased in both treatment groups at M6.5 (Figure 2D); though again as expected because this cohort received 4 higher doses of p55^Gag^ DNA vaccine, the T2 response rate was higher (Barnard’s test, *P* = 0.002).

In conclusion, the vaccine regimen used in T1 showed that the p24CE DNA vaccine was immunogenic and that both CD4^+^ and CD8^+^ T cell responses were increased after the combined (CE+gag) booster vaccinations. In contrast, the T2 regimen with full-length p55^Gag^ DNA–only vaccination resulted in lower CE responses, which were not increased even after the full set of 4 vaccinations. Together, these results were consistent with preclinical studies in nonhuman primates (45, 46).

### Increased CE-specific T cell response breadth in T1.

**Figure 3:**
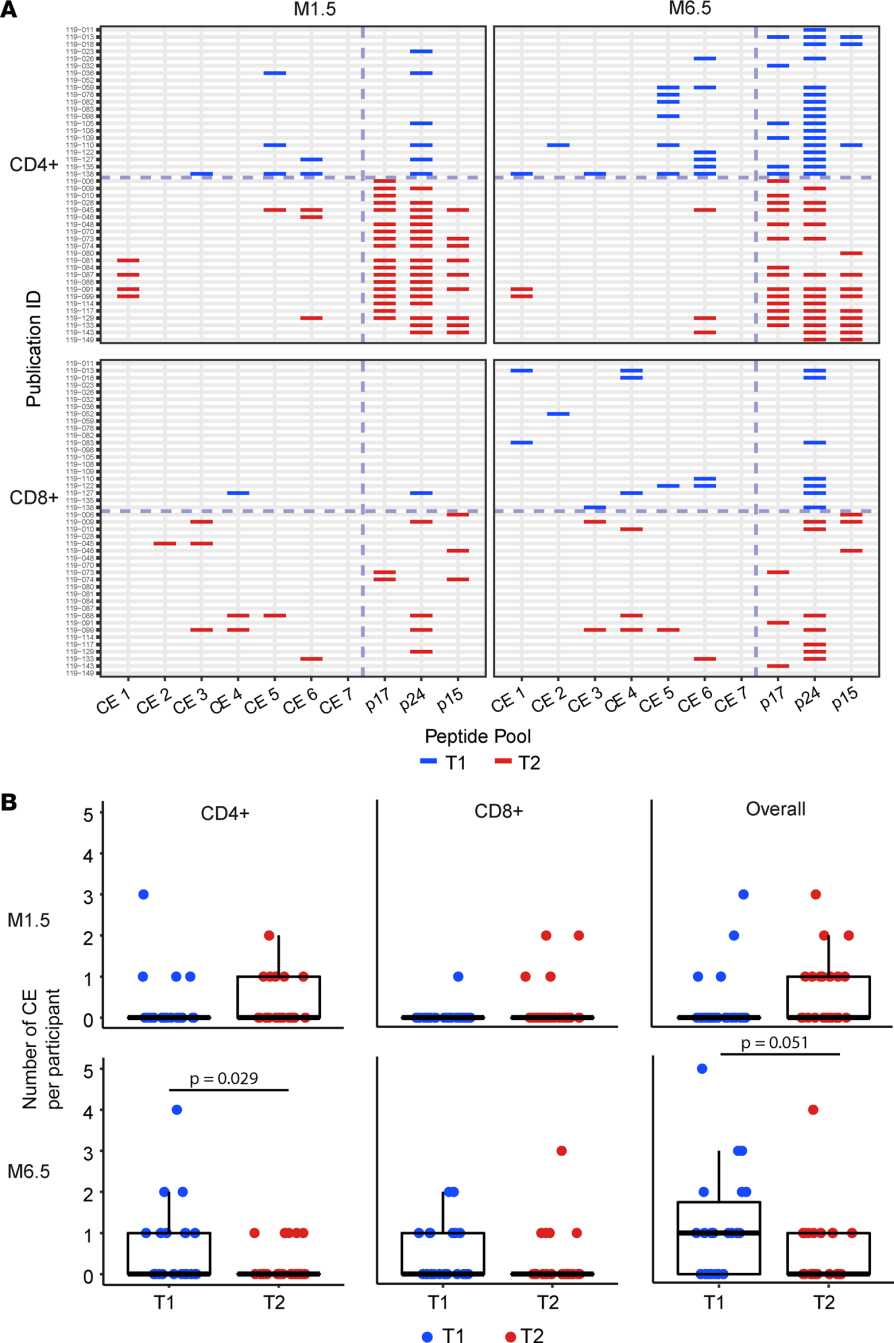
T cell responses to individual CE and Gag regions. (**A**) Drawing depicts participant-level mapping of positive responses by T cell subset (CD4^+^, CD8^+^), peptide pools, visit (M1.5 and M6.5), and treatment group (T1, T2). Responses to individual CE (CE 1 to CE 7) and p17^Gag^, p24^Gag^, and p15^Gag^ regions are shown for each study participant. (**B**) Box plots of breadth (number of CE recognized per participant) by T cell subset (and overall), visit, and treatment (see Methods).

**Figure 4:**
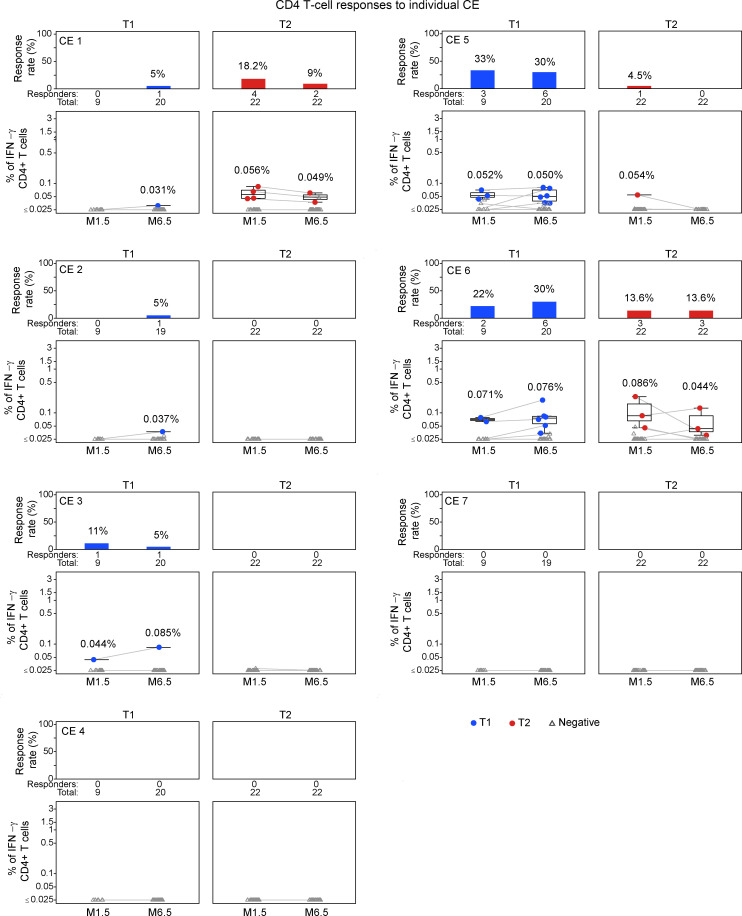
Mapping CD4^+^ T cell responses to individual CE. Flow cytometric analysis of individual CE. Each CE peptide pool comprised mixtures of 15-mer peptides overlapping by 11 AA and 10-mer peptides overlapping by 9 AA. IFN-γ CE-specific CD4^+^ T cell responses in the T1 and T2 groups are shown. Median response rates and magnitudes of positive values are shown. The bar (based on Fisher’s response; see Methods) and line plots show the distribution of response rates (upper panels) and the magnitude (lower panels), respectively, of individual CE-specific responses at M1.5 and M6.5.

**Figure 5:**
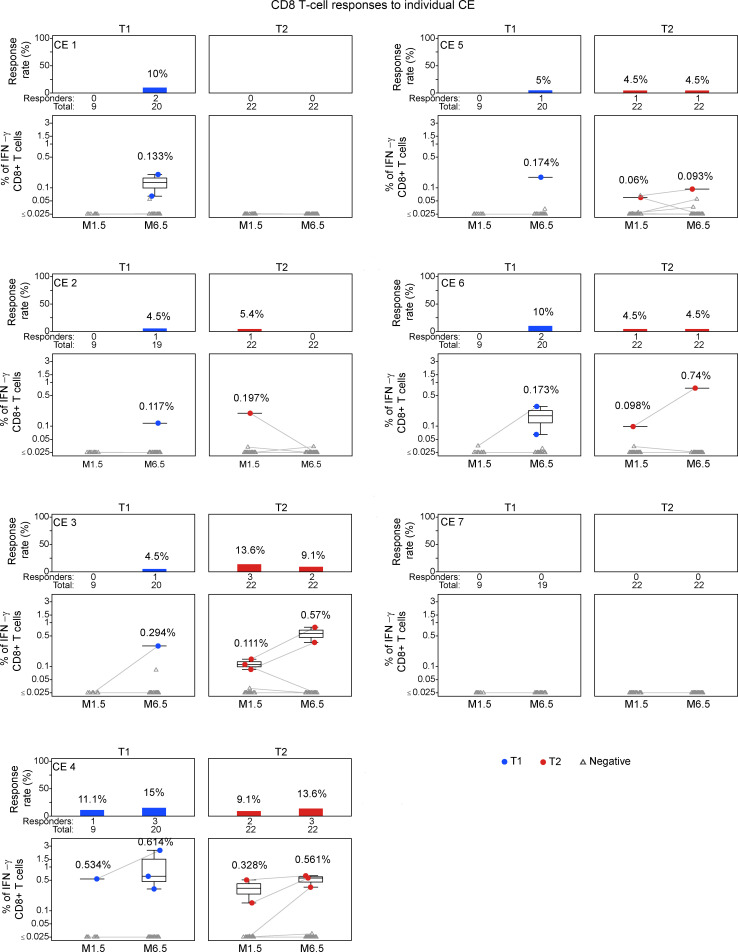
Mapping CD8^+^ T cell responses to individual CE. Flow cytometric analysis of individual CE was performed as described for Figure 4. IFN-γ CE-specific CD8^+^ T cell responses in the T1 and T2 groups are shown.

We next evaluated responses to the 7 individual CE peptide pools for participants with positive T cell responses (see Figure 2). An overview of the CD4^+^ and CD8^+^ T cell responses to 7 individual CE from each participant (T1, *n* = 23; T2, *n* = 21) is in the landscape display in Figure 3A. The breadth of the response, expressed as number of positive CE responses in each participant, is shown in Figure 3B. The response rates and magnitudes for individual CE peptide pools in T1 and T2 are shown in Figures 4 and 5.

At visit M1.5, 4 participants in T1 and 7 in T2 had CD4^+^ T cell responses that were detectable to at least 1 CE region, compared with 10 and 5 participants, respectively, at the M6.5 visit (Figure 3A). A similar trend toward increasing responses at M6.5 was noted for CD8^+^ T cell responses, with T1 increasing from 1 to 8 and T2 remaining at 5. By M6.5, T1 showed CD4^+^ T cell responses detected against 5 of 7 CE, while T2 had responses against only 2 CE (CE 1 and CE 6) (Figure 3A and Figure 4). Thus, the CE prime–CE+p55^Gag^ DNA boost vaccine regimen in T1 induced overall a broader CE response, resulting in more participants with detectable CE responses.

Analysis of individual CE responses showed that at M6.5, CD4^+^ T cell response rates were significantly higher in T1 for CE 5 (30% in T1 vs. 0% in T2; *P* = 0.006, Barnard’s test). CE 6 response was also higher (30% in T1 vs. 13.6% in T2; *P* = 0.239), though it did not reach significance. CD8^+^ T cell response rates to the individual CE peptide pools (Figure 4) were lower across both treatment arms, as expected from the analysis with the total CE pool (Figure 2). In contrast with CD4^+^ T cell response patterns, CD8^+^ T cell responses were detected against CE 4, while CE 7 failed to elicit CD4^+^ or CD8^+^ T cell responses (Figure 5). We hypothesized that inclusion of strictly CE for which no epitopes had been defined might reveal otherwise highly subdominant responses; however, there was no clear indication of this (Table 4), and no responses were detected against CE 7.

The breadth of CE responses, presented as the number of CD4^+^, CD8^+^, and overall (total number of positive CD4^+^ or CD8^+^ CE) in T1 and T2, is in Figure 3B. Statistical analysis was then applied to compare response breadth; if no response was detected, breadth was assigned a value of 0. At M1.5, CD4^+^- and CD8^+^-specific T cell breadths showed a maximal number of 3 and 1 CE for T1, respectively, with a similar response (maximal 2 CE for T2) (Table 5). At M6.5, the CD4^+^ T cell breadth was higher in T1 (maximum 4 CE) then T2 (maximum 1 CE), with a statistically significant difference of 0.46 CE and *P* = 0.029 (likelihood ratio test) (Table 6 and Figure 3B). The CD8^+^ T cell breadth at M6.5 was similar across treatment arms (T1: maximum 2 CE; T2 maximum 3 CE) (Table 5). Comparison of overall (CD4^+^ and or CD8^+^) T cell breadth between treatment arms at M6.5 showed a strong trend favoring T1 (T1: mean 1.136 CE, range 0–5 CE; T2 mean 0.52 CE, range 0–4 CE), with a difference of 0.615 CE showing a trend of significance with *P* = 0.051 (Table 6 and Figure 3B).

### Mapping T cell responses to individual Gag regions.

**Figure 6:**
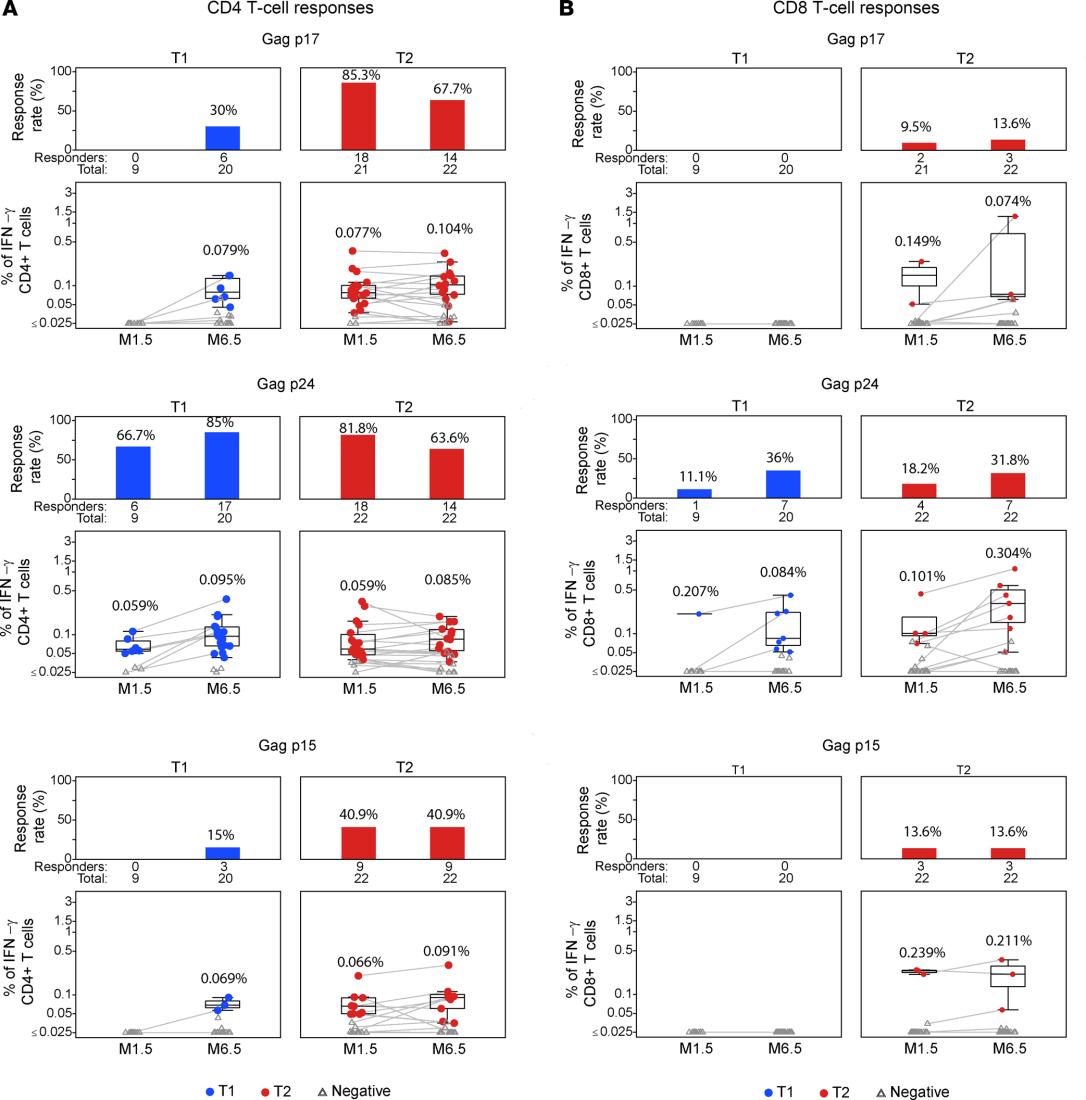
T cell responses to the 3 proteolytic cleavage products p17^Gag^, p24^Gag^, and p15^Gag^ derived from p55^Gag^. Bar and line plots show T cell response rates (upper panels) and magnitudes (box-line plots, lower panels) at M1.5 and M6.5. IFN-γ^+^ p55^Gag^-specific CD4^+^ (**A**) and CD8^+^ (**B**) T cell responses to p17^Gag^, p24^Gag^, and p15^Gag^ were measured at M1.5 and M6.5 in the T1 and T2 groups. Peptide pools comprised a mixture of 10-mer peptides overlapping by 9 AA spanning each protein. Response rates are given as the percentage of all vaccine recipients analyzed. Median response magnitudes are given in the panels as percentage of CD4^+^ and CD8^+^ T cells found in positive responders (see Methods).

We next mapped the responses to the 3 Gag peptide pools (Figure 3A and Figure 6) comprising the proteolytic products of Gag found in virions, including p17^Gag^, p24^Gag^, and p15^Gag^ (see Figure 1). As noted for the CE-specific responses above, CD4^+^ T cell response rates were also higher (Figure 6A) than CD8^+^ T cell response rates (Figure 6B) in both treatment groups for the different Gag peptide subpools.

The response rate to p24^Gag^ (the only Gag protein containing CE) at M6.5 was similar between groups (85% for T1 vs. 64% for T2). Responses to p17^Gag^ and p15^Gag^ increased from 0% to 30% (Barnard’s test *P* = 0.034) at M6.5 in T1, whereas T2 had CD4^+^ and CD8^+^ T cell responses to both proteins at both M1.5 and M6.5. CD4^+^ T cell response rates against p17^Gag^ (85%) and p15^Gag^ (41%) were higher in T2 at M1.5 than T1 at M6.5 (p17^Gag^, 30%; *P* < 0.001; p15^Gag^, 15%; *P* = 0.04; Barnard’s 1-sided test). The CD8^+^ T cell response rates to the 3 Gag peptide pools ranged from 0% to 38% for T1 and 10% to 32% for T2. These data suggest that the immunodominance favoring CE afforded by the T1 regimen persisted through the booster regimen, with the caveat that the T1 group received a lower dose of p55^Gag^ DNA vaccine.

In T2, only 5 of the 14 individuals with responses to p24^Gag^ had responses to CE at M6.5 (Figure 3A), showing that the responses were mounted against epitopes outside the CE in the majority of participants. In T1, by contrast, 10 of 17 individuals at M6.5 had responses to CE, while only one-third of participants had responses directed only to epitopes outside of CE within p24^Gag^ (Figure 3A).

The magnitude of the responses among the positive responders paralleled the response rates against p24^Gag^, reaching a median of 0.095% (maximum 0.369%) in T1 versus 0.085% (maximum 0.9%) in T2. Similarly, higher magnitudes were detected in T2 against p17^Gag^ and p15^Gag^ after 2 vaccinations compared with the T1 regimen.

### HIV-1 p24^Gag^ antibody responses.

**Figure 7:**
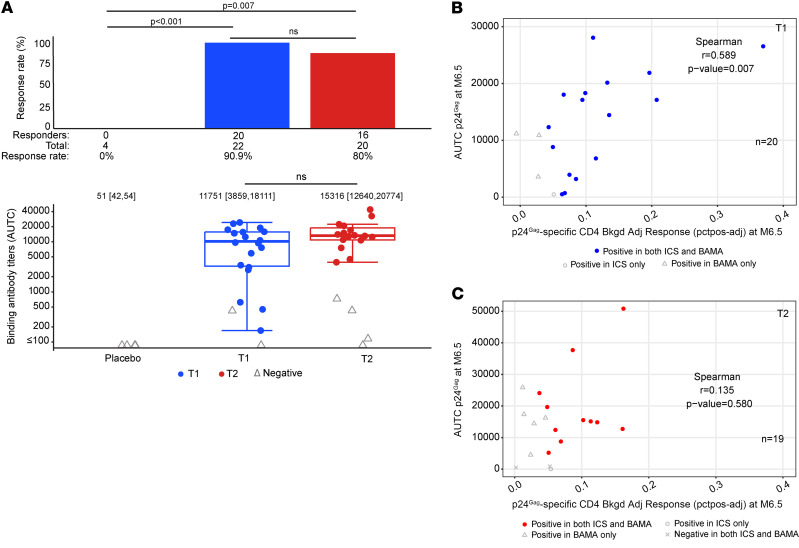
p24^Gag^ antibody responses. The binding antibody multiplex assay (BAMA) to p24^Gag^ was performed at M6.5. (**A**) Response rates (upper panel) listing number of responders, number of samples analyzed, and response rate in percentage points. *P* values are from Wilcoxon rank-sum tests. The lower panel shows the area under the curve (AUTC) from responders in color and nonresponders in gray with box plots based on data from responders superimposed on the distribution. The midline of the box denotes the median, and the boundaries of the box denote the 25th and 75th percentiles. The whiskers extend to the most extreme data points that are no more than 1.5 times the interquartile range (i.e., height of the box) or, if no value meets this criterion, to the data extremes. (**B** and **C**) Correlation between p24^Gag^ antibody level (AUTC, from panel **A**) and p24^Gag^-specific IFN-γ^+^ CD4^+^ T cell response at M6.5 in (**B**) T1 and (**C**) T2 (data from Figure 6). Spearman’s *r* and *P* values are given.

p24^Gag^-specific IgG levels were measured using the BAMA on sera collected at M6.5 (Figure 7). The response rates to p24^Gag^ were not significantly higher in T1 (91%) versus T2 (80.0%) (Figure 7A) (Barnard’s test, *P* = 0.36). Among positive responders, the median AUTC was nonsignificantly higher in T2 (median AUTC 15,316) than T1 (median AUTC 11,751) (Wilcoxon rank sum test *P* = 0.149) (Figure 7A).

Interestingly, p24^Gag^ antibody AUTC and p24^Gag^-specific CD4^+^ T cell responses showed a strong positive correlation (Spearman’s *r* = 0.589, *P* = 0.007) for T1 (CE vaccine; *n* = 20) (Figure 7B) but not T2 (*n* = 10; Spearman’s *r* = 0.155, *P* = 0.654) (Figure 7C). This suggests the CE immunogen also enhances antibody responses to CE, agreeing with results obtained in macaques (59).

## Discussion

HVTN 119’s aim was to test whether a DNA vaccine regimen focuses immune responses toward regions of the p24^Gag^ protein that are invariable or functionally critical across Group M HIV (i.e., CE) in adults without HIV. Consistent with preclinical studies in mice and rhesus macaque models (44–46, 59), vaccination with CE+p55^Gag^ (T1) was superior to p55^Gag^ DNA only (T2) in focusing immune responses on conserved regions within p24^Gag^, resulting in greater response rate and significantly increased breadth.

Our study draws comparison to another DNA vaccine study using EP for DNA delivery (HVTN 087) (51, 53). The latter study tested immunogenicity of a Gag/Pol DNA vaccine including the same IL-12 DNA as adjuvant, the same IL-12 DNA dose, and the same DNA delivery device and using the same flow cytometry assays. Overall, CD4^+^ T cell responses were greater than CD8^+^ T cell responses in both trials. Although HVTN 087 showed a benefit in increasing Gag-specific CD8^+^ T cell response upon inclusion of IL-12 DNA as adjuvant, the responses remained overall skewed toward CD4^+^ T cells. In HVTN 119, using the same IL-12 DNA as adjuvant, the outcome in CD4^+^ and CD8^+^ responses was consistent with HVTN 087. Perhaps more local production of IL-12 at the time of vaccination with a more efficient plasmid cytokine would further increase cellular responses and increase the ratio of CD8^+^ to CD4^+^ T cell responses (47, 60). Other cytokine adjuvants, in either protein or plasmid form, could also augment vaccine responses. Administration of recombinant IL-7 subcutaneously in macaques resulted in dramatic changes within lymph nodes, including enlarged germinal centers, increased IL-21 production, and expansions of B cells and CD4^+^ and CD8^+^ T cells (61). However, recombinant IL-7 has thus far only been tested in cancer immunotherapy trials in humans (62). It is also possible that using liposomal nanoparticles, as used for the SARS-CoV-2 mRNA vaccine in humans (63–65), which rapidly activates the innate immune system, may bring about this type of advantage.

Boosting with recombinant viral vector, i.e., recombinant vesicular stomatitis virus in HVTN 087, increased T cell responses but did not alter the CD4/CD8 ratio of antigen-specific T cell responses (53). Of note, studies in nonhuman primates showed a stronger skewing of responses toward CD8 (45, 46), and thus, in this aspect, these studies were less predictive of the outcome in HVTN 119.

In addition, results from our p55^Gag^ DNA arm (T2; 4 mg) compared with HVTN 087 (3 mg Gag/Pol DNA) showed remarkable differences in response rate and magnitude. HVTN 119 resulted in higher CD4^+^ response rates (79% at M1.5 and 71% at M6.5) compared with HVTN 087 (19% at M3.5, 2 weeks after the third DNA vaccination) and CD8^+^ response rates (40% at M1.5 and 70% at M6.5 versus 5% at M3.5 in HVTN 087). HVTN 119 also showed a higher magnitude of IFN-γ or IL-2 CD4^+^ and CD8^+^ T cell responses. The superior immunogenicity of the DNA vaccine in the current study may result from improved Gag expression and vector design. The vector employed here eliminated all splice sites and enhanced transcription, nucleocytoplasmic transport, and translation of mRNA (44).

Dissection of CE-specific responses, induced by the 2 vaccine regimens at M6.5, showed the CE regimen induced significantly more CD4^+^ T cell responses to CE, with 10 participants showing responses to 1 to 4 CE, whereas the p55^Gag^ DNA vaccine induced responses to 1 CE in 5 participants. The CD8^+^ T cell responses were more similar among the groups (T1, 8 participants with 1 to 2 CE; T2, 5 participants with 1 to 3 CE). Overall, we found stronger CD4^+^-mediated response in people without HIV, whereas the same vaccine induced more CD8^+^ responses in PLWH receiving antiretroviral therapy (55). This could reflect higher preexisting CD8^+^ T cell responses, as observed (66). Comparing the overall responses, we found in T1, 14 participants had responses to 1 to 5 CE, with 6 participants showing responses to more than 1 CE (3 with 2 CE; 2 with 3 CE; 1 with 1 CE). In contrast, in T2, 7 participants had responses ranging from 1 to 4 CE, with only 1 participant showing responses to more than 1 CE (i.e., CE 5). These data showed a strong trend (*P* = 0.051) of overall increased breadth (CD4^+^ and or CD8^+^). In conclusion, HVTN 119 showed that the CE prime–CE+p55^Gag^ DNA boost vaccine induced T cell responses to more CE than the classical p55^Gag^ DNA vaccine.

A larger number of participants in T2 developed CD4^+^ T cell responses to p24^Gag^ after 2 immunizations, compared with T1 after 4 immunizations that included only 2 vaccinations with p55^Gag^ DNA. This could have been due to the lower amount of p55^Gag^ DNA delivered to the T1 group (2 mg in T1 vs. 4 mg in T2), which was necessary to deliver equivalent amounts of total DNA in each dose. However, establishment of an altered immunodominance hierarchy focused on CE may also have contributed to this result. Consistent with this possibility was the finding that the booster vaccinations that included p55^Gag^ DNA in T1 increased responses to CE well above those observed in T2.

Alternative approaches to CE/CE+p55^Gag^ such as the use of complete p55^Gag^ immunogen with mutated known immunodominant epitopes were not tested. This approach was tested in an influenza nucleoprotein/mouse model (67) and induced a protective T cell response against cryptic epitopes. The success of such an approach may depend on the protein and mutations introduced. In addition to inducing T cell immunity, the CE DNA vaccine regimen induced strong anti-p24^Gag^ antibody responses, as previously noted in the rhesus macaque model (59). Interestingly, a significant correlation was observed here between p24^Gag^-specific CD4^+^ T cell responses and p24^Gag^ antibody levels, suggesting a role for CD4^+^ T cell help in the development of Gag-specific antibody (68). While not performed as part of the primary analysis of this study, we suspect the robust CD4^+^ responses induced by this vaccine regimen could include activation of circulating T follicular helper cells as seen in prior HVTN studies (69, 70). Such comprehensive analyses will be integral to understanding the ability of complex vaccination regimens to elicit coordinated cellular and humoral immune responses.

In a recent parallel study, the same vaccine regimens employed here were tested in PLWH receiving antiretroviral therapy (55). That study showed the CE DNA vaccine regimen can partially overcome preexisting immunodominance to induce new CE-directed cellular immune responses. Thus, while the elicited immune responses were modest in both studies, CE/CE+p55^Gag^ DNA or similar regimens could provide valuable approaches for focusing immune responses in naive or preexisting immunity.

EP has been shown to dramatically increase the immunogenicity of DNA vaccines delivered IM (49) and after intradermal vaccination and can elicit robust cellular and humoral immune responses (53). However, the particular device used may have had an important impact on the delivery and tolerability of vaccination. Most of the errors in HVTN 119 occurred at the start of the trial but were distributed across all 3 sites. The rate of errors appeared to decrease with a new lot of needle arrays and extra staff training from the manufacturer, but ultimately there was no specific cause identified. Despite these device difficulties, the HVTN 119 vaccine regimen led to robust cellular and humoral immune responses, and we suspect the differences found between active study arms would have been even greater with more efficient EP.

While the described p24CE vaccine and boosting with CE+gag also induced immune responses to CE and epitopes outside the highly conserved regions, this regimen should be considered as part of a combinatorial vaccine that includes a regimen inducing broadly neutralizing antibodies. We hypothesize that this vaccine concept may have further application in the development of vaccines of greater potency against HIV and other pathogens as well as in other fields, such as targeted cancer immunotherapy.

## Methods

### Sex as a biological variable

The study design did not account for sex as a biological variable, and details on the study cohort are provided in Table 1.

### Group size and statistical analysis

The sample size of *n* = 25 was based on the primary and secondary endpoint comparisons of CD4^+^ and CD8^+^ T cell breadth to the CE peptides. Power was calculated for sample sizes of *n* = 20, 25, and 30 assuming 17% missing data. The T cell breadth endpoints assumed a β-binomial distribution, and the ICS endpoint assumed normally distributed log-transformed magnitudes with variance based on data from the HVTN 080 trial (49).

A sample size of *n* = 25 was selected based on the effect size necessary for 80% power to detect a difference between the 2 groups under 2 scenarios for the breadth comparison and a fold-change in the magnitude of ICS response. The calculated effect size to detect a difference in CE breadth, given a mean breadth of 0.2 (or 0.5) in group T2, was a breadth of 1.0 (1.5) or greater in group T1. The calculated effect size to detect a difference in ICS magnitude was a 2.5 fold-change or greater increase in magnitude in group T1.

### Participant cohort

HVTN 119 completed enrollment on October 31, 2018, with 56 participants, ages 28–50 years: 25 in T1, 25 in T2, and 6 in the control arm. A total of 46 participants (22 in T1 and 24 in T2) met the sampling criteria specified in the statistical methods section to be further assayed against the 7 CE total and 3 p55^Gag^ peptide pools. Among those participants, 32 had positive responses at M1.5 and M6.5, 4 were positive at only M1.5, and 10 were positive at only M6.5. This resulted in a total of 78 samples with T cell epitope mapping data.

The sample selection was based on IFN-γ or IL-2, and on CD40L responses, but breadth was based on IFN-γ responses; 1 sample for T1 that was positive to CE was missing T cell epitope mapping data and was excluded from the analysis. As specified in the “Flow cytometry” section, numbers reported here do not reflect additional filtering at the peptide pool and T cell subset level.

### DNA vaccine study agents

#### p24CE1/2.

p24CE1/2 DNA (plasmid 306H) encodes the 2 CE variants CE1 and CE2. The p24CE1 and p24CE2 genes were produced synthetically and were inserted into the eukaryotic expression vector pDP.CMVkan, a derivative of pVR1012 (71). The p24CE1 gene is expressed from the huCMV promoter and is terminated by the BGH polyA signal. The p24CE2 gene is expressed from the siCMV promoter and is terminated by the SV40 polyA signal. The complete sequences for p24CE1 and p24CE2 code for 157 AA each (127 AA spanning CE 1 to CE 7, 13 AA linker sequences, 17 AA GM-CSF signal peptide) (44) are provided in Supplemental Table 1. The p24CE proteins encompass 54% of p24^Gag^ and 25% of the full-length p55^Gag^ protein. The 17-AA GM-CSF signal peptide (GenBank: AAA98768.1) favorably affects localization and stability of p24CE1 and p24CE2 proteins as well as the nature of elicited immune responses (44). The sequences of both genes were optimized to enhance expression in human cells (44).

#### p55^Gag^ DNA.

The p55^Gag^ DNA (plasmid 114H) spans 5,518 bp and encodes the full-length p55^Gag^ protein of HIV-1 (clade B isolate HXB2; GenBank NP_057850.1), consisting of 500 AA spanning the proteolytic processing products p17^Gag^, p24^Gag^, and p15^Gag^. The expression-optimized p55^Gag^ gene was chemically synthesized and inserted into the expression plasmid pCMVkan under the control of the huCMV promoter and terminated by the BGH polyA signal. The pCMVkan plasmid backbone is derived from pVR1012 (71).

#### IL-12 DNA.

The human IL-12 DNA (GENEVAX IL-12 DNA plasmid; Profectus BioSciences) spans 6,259 bp and encodes the IL-12 p35 and p40 proteins under separate regulatory control in counterclockwise orientation from a dual-promoter plasmid. This plasmid was also used in HVTN 060, 063, 070, and 080 clinical protocols (49–51, 54). The p35 subunit is expressed from the huCMV promoter and terminated by the SV40 polyA, whereas the p40 subunit is expressed from siCMV promoter and terminated by the BGH polyA; both genes are necessary to produce the heterodimeric bioactive IL-12 p70 protein within the same cell. The plasmid backbone contains the kanamycin resistance gene for selection in bacteria.

### Vaccines

Each drug product was formulated separately and administered via IM injection followed by EP. The p24CE1/2 DNA and p55^Gag^ DNA vaccines were formulated at 4 mg/mL with bupivacaine (0.25%) as a facilitating agent for DNA uptake and stabilization. The IL-12 DNA adjuvant was formulated at 2 mg/mL with bupivacaine (0.25%) and was mixed 1:1 with the DNA vaccines at the time of injection for coadministration. Placebo injections consisted of 0.9% sodium chloride, USP.

### Administration by EP

Both DNA vaccine regimens and the placebo were administered IM in the bilateral deltoid muscles using the intramuscular Ichor TriGrid Delivery System for in vivo EP (57, 58). Bilateral IM injections, in right and left deltoid muscles, were administered (1 mL per injection site) at each vaccination time point. Each study participant received up to the protocol-specified 4 doses over a 6-month period.

### Safety and tolerability assessments

Pre- and postvaccination, participants were assessed for systemic symptoms including increased body temperature, malaise and/or fatigue, myalgia, headache, chills, arthralgia, nausea, and vomiting; local symptoms of pain and/or tenderness and erythema and/or induration proximal to the injection site; and enlargement or tenderness of proximally draining lymph nodes. Pain related to EP was assessed using the VAS, and acceptability was assessed using a questionnaire. Social impact of study participation was also assessed.

### Flow cytometry

Flow cytometry was used to examine HIV-1–specific CD4^+^ and CD8^+^ T cell responses using a validated ICS assay. A 28-color panel was used for the initial testing of total CE and p55^Gag^ peptide pools, and a 17-color panel (experiment Assay ID 109; analysis plan 042) was used for the mapping to individual CE regions and subregions of p55^Gag^ (72, 73) (Supplemental Tables 3 and 4).

The peptide pools evaluated included total CE and 7 individual CE peptide pools (CE 1 through CE 7). The CE pools comprised a mixture of 15-mer peptides overlapping by 11 AA and 10-mer peptides overlapping by 9 AA spanning all CE and each individual CE. The HXB2 p55^Gag^ pool comprised 15-mer peptides overlapping by 11 AA. The p17^Gag^, p24^Gag^, and p15^Gag^ pools comprised mixtures of 15-mer peptides overlapping by 11 AA and 10-mer peptides overlapping by 9 AA spanning each region.

Cryopreserved specimens were stimulated with synthetic peptide pools. As a negative control, cells were left unstimulated, except for inclusion of DMSO, the peptide diluent. As a positive control, cells were stimulated with a polyclonal stimulant, staphylococcal enterotoxin B. There were no replicates except for the negative control, which had 2 replicates. As an additional internal control, cells were stimulated with a CMV pp65 peptide pool.

Antigen-specific responses were measured for CD4^+^ T cells expressing CD40L, IFN-γ and/or IL-2, or IFN-γ and CD8^+^ T cells expressing IFN-γ and/or IL-2 or IFN-γ. Responses to individual CE were reported for IFN-γ rather than IFN-γ and/or IL-2 since the IFN-γ measurement was somewhat more sensitive (likely due to higher background introduced by IL-2). Analysis details are provided in Supplemental Methods.

### Statistics

#### Breadth.

For participants with a positive response to total CE or p55^Gag^ for either IFN-γ and/or IL-2 (CD4^+^ or CD8^+^ T cells) or for CD40L (CD4^+^) at M1.5 and/or M6.5, samples were assayed against the 7 individual CE peptide pools, and the total number of positive responses was defined as the breadth. Breadth was calculated separately for each T cell subset and time point. Overall T cell breadth was defined as the sum of CD4^+^ and CD8^+^ T cell breadths. Breadth was determined by first measuring an IFN-γ response to total CE; breadth summaries include all vaccine participants with ICS 27-color panel results. If no response was detected, then the breadth was assigned a value of 0.

Fisher’s response rates with corresponding 95% confidence intervals were calculated by the score test method (74). Barnard’s test was used to compare response rates between treatment arms (T1 vs. T2) (75). Negative binomial regression was used to calculate point and 95% profile likelihood-based confidence interval estimates of mean breadth by T cell subset and overall, for each treatment arm (T1 and T2) and for mean differences between treatment arms. Likelihood ratio tests from these models were used to obtain *P* values for testing whether mean breadth differed between treatment arms. For all comparisons, *P* values are 2 sided and unadjusted for multiplicity, with significant differences declared if *P* ≤ 0.05. The distribution of the background-adjusted magnitude of IFN-γ^+^ T cell response was displayed graphically on the log scale by T cell subset, peptide pool, visit, and treatment arm.

#### Gag antibody measurements.

Serum HIV-1–specific IgG antibody responses were measured against the clade B p24^Gag^ antigen on a Bio-Plex instrument (Bio-Rad) using a standardized custom HIV-1 Luminex assay (BAMA) (76, 77). Response rates were determined at a 1:50 serum dilution. Antibody titers were measured by serum titration starting at 1:500 followed by a 10-fold serial dilution for a total of 4 dilution points, allowing MFI values to fall within the linear range of the assay and AUTC to be calculated. The readout was background-subtracted MFI, where background referred to a plate-level control (i.e., a blank well run on each plate). Standard positive and negative controls were included in each assay to ensure specificity and for maintaining consistency and reproducibility between assays. The positive control included purified polyclonal IgG from HIV individuals using a 10-point standard curve (4-parameter logistic regression fit). The negative controls were NHS (HIV-1–seronegative human sera) and blank beads. To control for protein performance, the preset criteria include that the positive control titration in each assay had to be within ±3 SEM for each antigen (tracked with a Levey-Jennings plot for high MFI and AUTC). Four participants (2 in placebo, 2 in T2) were filtered because of high blank (>5,000 MFI) at sample or baseline visits. One participant in T2 was filtered because of high MFI minus blank (>6,500) at baseline. One participant in T1 was filtered because of blood draw date being outside the allowable visit window.

#### BAMA.

Samples were declared to have positive responses if they met 3 conditions: 1) MFI minus blank (MFI*) values ≥ antigen-specific cutoff at 1:50 dilution level (based on 95th percentile of baseline samples as calculated by SAS PROC UNIVARIATE default method, and at least 100 MFI minus blank), 2) MFI minus blank values > 3 × baseline (day 0) MFI minus blank values, and 3) MFI values > 3 × baseline MFI values. MFI minus blank values were truncated at 22,000, the upper limit of the linear range of the assay. Samples were excluded from threshold calculation and analysis if the baseline analyte MFI minus blank exceeded 6,500. For each combination of individual and antigen the AUTC at M6.5 was calculated over dilutions 500, 5,000, 50,000, and 500,000 using the trapezoidal integration, with log_10_ transformation of the dilutions and with truncation at 0 in the case of negative net MFI values.

Response rates and corresponding 95% confidence intervals were calculated by the Wilson score method (74). Barnard’s test was used to compare response rates between pairs of groups, while the Wilcoxon rank sum test was used to compare AUTC response magnitudes among positive responders between pairs of groups. Group comparisons include T1 versus control, T2 versus control, and T1 versus T2. The *P* values are 2 sided and unadjusted for multiple comparisons. *P* < 0.05 was considered significant.

### Study approval

The study was approved by the following institutional review boards: Emory University (Atlanta, Georgia, USA; 00097680); Cleveland University Hospitals (Cleveland, Ohio, USA; approval 07-17-12); and UCSF (approval 17-22399). Written and oral informed consent were provided by all participants before inclusion.

### Data availability

Values for all data points found in graphs are in the Supporting Data Values file, and data are available at https://atlas.scharp.org/cpas/project/HVTN%20Public%20Data/HVTN%20119/begin.view

## Author contributions

BKF, GNP, and JIM conceptualized the study. SAK, MNP, DH, MAA, JE, MAE, and HMS contributed to the concept, design, clinical product development, and implementation of this clinical trial. SAK, MAA, and HMS contributed to the medical monitoring and study oversight. BKF, GNP, JIM, MR, and JB contributed to the design and preliminary evaluation of the study products. SCDR, MJM, and GDT contributed to the immunogenicity testing. SCDR, ACD, J Heptinstall, and GDT performed formal analysis. J Hu and ACD performed the statistical analysis. BKF, JIM, GNP, and SAK wrote the original and the final manuscripts. All authors contributed to the review and editing of the manuscript.

## Supplementary Material

Supplemental data

ICMJE disclosure forms

Supporting data values

## Figures and Tables

**Table 1:**
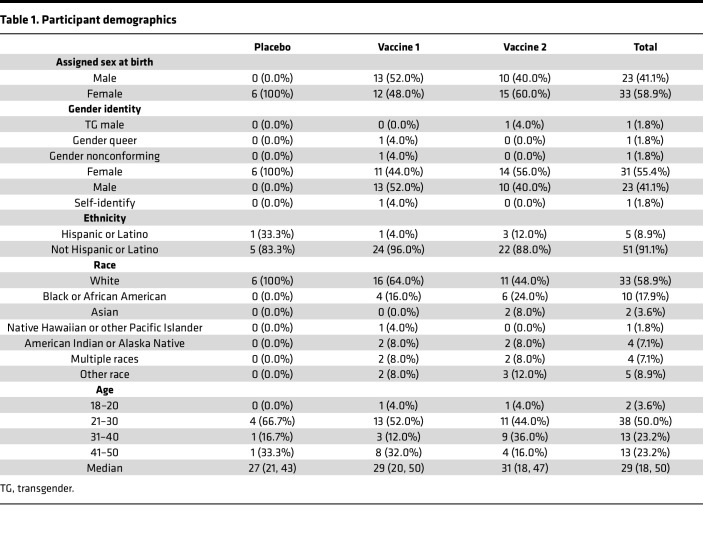
Participant demographics

**Table 2:**
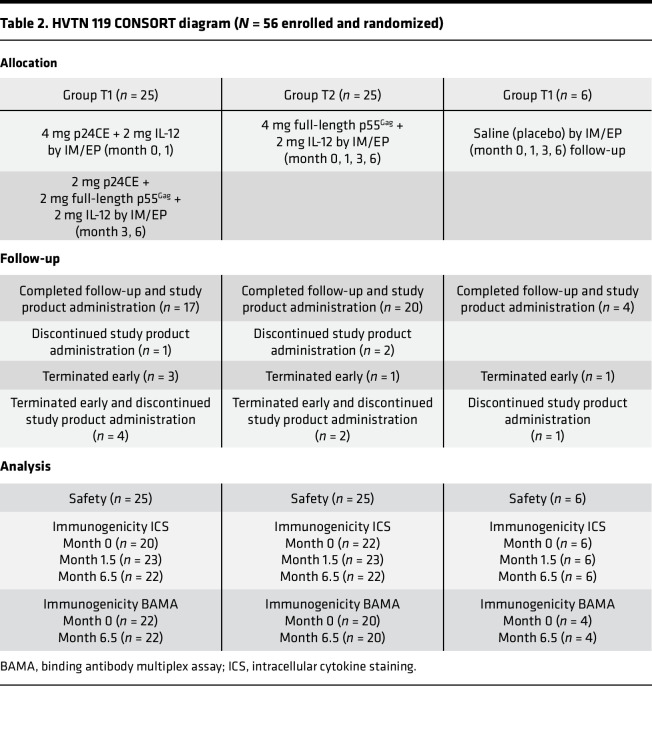
HVTN 119 CONSORT diagram (*N* = 56 enrolled and randomized)

**Table 3:**
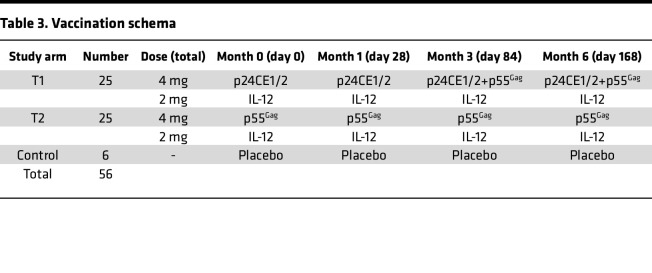
Vaccination schema

**Table 5:**
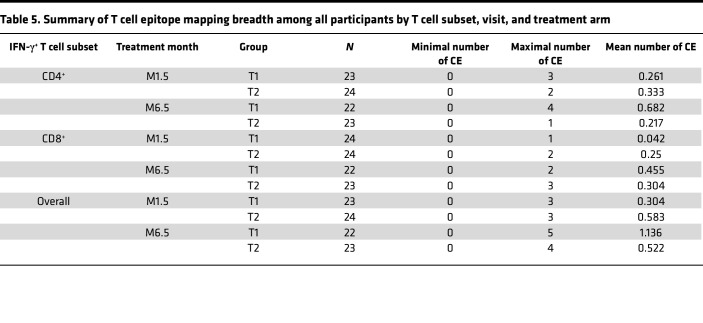
Summary of T cell epitope mapping breadth among all participants by T cell subset, visit, and treatment arm

**Table 6:**
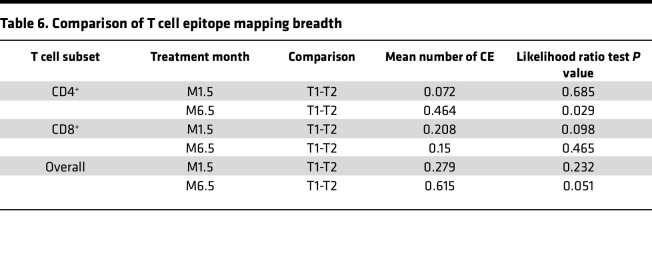
Comparison of T cell epitope mapping breadth

**Table 4:**
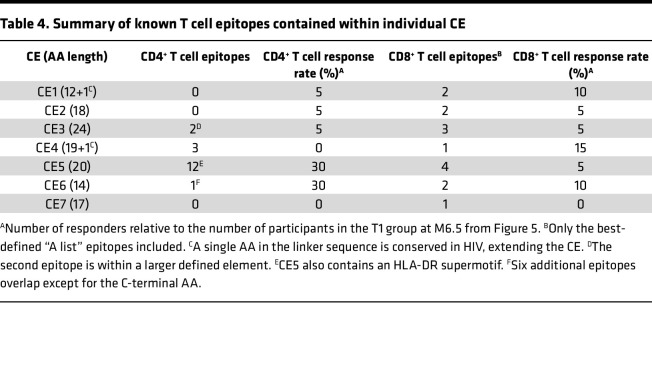
Summary of known T cell epitopes contained within individual CE

## Version 1 08/01/2024

In-Press Preview

## Version 2 09/24/2024

Electronic publication
