# Supplementary material for: Focusing HIV-1 Gag T cell responses to highly conserved regions by DNA vaccination in HVTN 119
Source: JCI Insight. 2024 Aug 1;9(18):e180819. doi: 10.1172/jci.insight.180819 (PMC11466283; doi:10.1172/jci.insight.180819)
Supplement: Supplemental data [file jciinsight-9-180819-s014.pdf]

## Supplemental Materials and Methods

*ICS Criteria*

Several criteria were used to determine if data from an assay were acceptable and could be statistically analyzed. The blood draw date must have been within the allowable visit window as determined by the protocol. After sample thawing and overnight incubation, the viability of the PBMCs must have been 66% or greater. If it was not, a new specimen for that participant at that time point was thawed for testing. If the PBMC viability of the second thawed aliquot was below this threshold, the ICS assay was not performed, and no data were reported to the statistical center for the participant and time point. For the negative control acceptance criteria, if the average cytokine response for the negative control wells was above 0.1% for either the CD4+ or CD8+ T cells, the sample was retested. If the retested results were above 0.1%, the data were excluded from analysis; otherwise, the retest data were used. The total numbers of CD4+ and CD8+ T cells must also have exceeded certain thresholds. If the number of CD8+ T cells was less than 5,000 or CD4+ T cells was less than 10,000 for any of the HIV-1 peptide pools or for one of the negative control replicates for a particular sample, data for that stimulation were filtered. If both negative control replicates failed for the number of T cells, the sample was retested. If 1 negative control replicate failed for number of T cells, the negative control replicate with sufficient T cells was used.

*ICS assay*

To assess positivity for a peptide pool within a T cell subset, a 2-by-2 contingency table was constructed comparing the HIV-1 peptide-stimulated and negative control data. The 4 entries in each table were the number of cells positive for IFN-γ and the number of cells negative for IFN-γ, for both the stimulated and the negative control data. If both negative control replicates were

included, then the average number of total cells and the average number of positive cells were used. A 1-sided Fisher's exact test was applied to the table, testing whether the number of cytokine-producing cells for the stimulated data was equal to that for the negative control data. Typically, multiplicity adjustment was made to the individual peptide pool P values since multiple individual tests (for each peptide pool) were conducted simultaneously; this was not done for mapping studies to provide higher sensitivity to detect responses. If the P value for a peptide pool was no more than 0.00001, the response to the peptide pool for the T cell subset was considered positive. Because the sample sizes (i.e., total cell counts for the T cell subset) were large (up to 100,000 cells), the Fisher's exact test has high power to reject the null hypothesis for very small differences. The P value significance threshold was chosen stringently (≤0.00001).

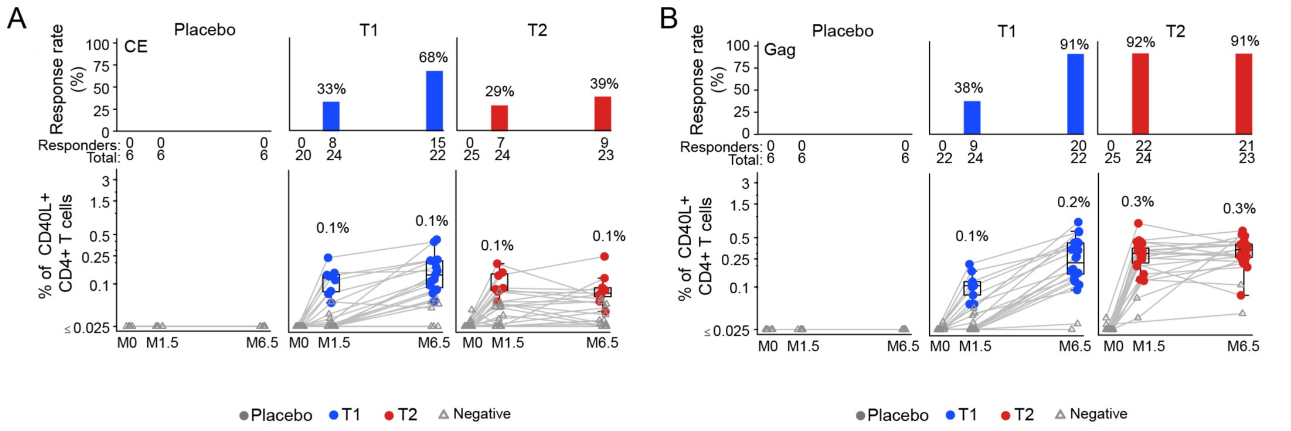


**Supplemental Figure 1. Flow cytometric analysis of CD40L$^+$ CD4$^+$ T-cell responses to CE and full-length p55$^{Gag}$.** CD40L$^+$ CD4$^+$ T-cell responses to (**A**) total CE and (**B**) p55$^{Gag}$ were measured in PBMC. Upper panels indicate the response rate (number of responders/number of participants tested). The lower panels show the distribution of background-adjusted magnitude of CD40L$^+$ T-cell responses are displayed graphically on the log scale y-axis truncated at 0.025%; any values below this level are censored. Plots include data from responders in color and non-responders in grey, with data points for each participant are connected by a grey line. Box plot whiskers extend to the most extreme data points that are no more than 1.5 times the interquartile range (i.e., height of the box) or if no value meets this criterion, to the data extremes. Median response magnitudes are shown as % of CD40L$^+$ response.

**Supplemental Table 1. p24CE1 and p24CE2 proteins.**

```
                CE 2                       CE 3                          CE 4

p24CE1 VIPMFSALSEGATPQDLNAAVGGHQAAMQMLKDTINEEAAEWDRAAAEPRGSDIAGTTSTLQEQIGWAAA

p24CE2 VIPMFTALSEGATPQDLNAAVGGHQAAMQMLKETINEEAAEWDRAAAEPRGSDIAGTTSTLQEQIAWAAA


                CE 5                    CE 6                 CE 7              CE 1

p24CE1 KRWIILGLNKIVRMYSPTSIAAKYVDRFYKTLRAEQAAGLEEMMTACQGVGGPGHKAAISPRTLNAWVKV

p24CE2 KRWIILGLNKIVRMYSPVSIAAKYVDRFFKTLRAEQAAGLEEMMTACQGVGGPSHKAALSPRTLNAWVKV
```

Notes:
Differences between p24CE1 and p24CE2 are shown in red vs blue type, respectively.
Linker sequences are shown in green type.
Linker sequences that were conserved in HIV, when adjacent to CE are underlined.
CE 1 was placed at the C-terminus of each construct to enhance gene expression (Kulkarni V, et al. *PLos One.* 2013;8:e60245).

**Supplemental Table 2. Reason for missing ICS data.**

| | | | | | Data reported | | |
|---|---|---|---|---|---|---|---|
| Visit | Treatment | Expected n | Assayed n | Reason for unavailability | CD4 | CD8 | Reason missing |
| M0 | Placebo | 6 | 6 | - | 6 | 6 | - |
| | T1 | 25 | 22 | 1 no PBMC collected,<br>1 PBMC not shipped before assays performed,<br>1 no post-baseline PBMC (terminated) | 20 | 20 | 1 Low CD4 and CD8 T cell number, 1 CD4 and CD8 unreliable* |
| | T2 | 25 | 25 | - | 25 | 24 | 1 high CD8 background |
| M1.5 | Placebo | 6 | 6 | - | 6 | 6 | - |
| | T1 | 25 | 24 | 1 missed visit | 24 | 23 | 1 high CD8 background |
| | T2 | 25 | 24 | 1 no PBMC collected | 23 | 23 | 1 high CD4 and CD8 background |
| M6.5 | Placebo | 6 | 6 | - | 6 | 6 | - |
| | T1 | 25 | 22 | 2 missed visits, 1 out of window | 22 | 22 | - |
| | T2 | 25 | 23 | 1 missed visit<br>1 terminated | 21 | 23 | 2 high CD4 background |

*Missing both CD4 and CD8 only for Total CE; data for 22 available for $p55^{Gag}$

**Supplemental Table 3. Reagents in the 28-color ICS panel.**

| Antibody | Manufacturer | Catalog Number |
|---|---|---|
| UViD | Invitrogen | L34962 |
| CD3 BUV395 | BD Biosciences | 563546 |
| CD4 BV480 | BD Biosciences | 566165 |
| CD8a BUV805 | BD Biosciences | 564912 |
| CD45RA BUV496 | BD Biosciences | CUSTOM |
| CD154 (CD40L) BUV737 | BD Biosciences | 748963 |
| CD25 BUV563 | BD Biosciences | 565699 |
| TNF-α FITC | Invitrogen | 11-7349-82 |
| CD16 BV570 | BioLegend | 302036 |
| Perforin PE | BioLegend | 353304 |
| KLRG1 PE-Vio615 | Miltenyi | 130-108-366 |
| CXCR5 BV785 | BioLegend | 356936 |
| PD-1 BV650 | BioLegend | 329950 |
| IL-4 BB630[A] | BD Biosciences | CUSTOM |
| IL-13 BB630[A] | BD Biosciences | CUSTOM |
| HLA-DR BUV661 | BD Biosciences | 565073 |
| CD14 BB660 | BD Biosciences | 624295 |
| CTLA-4 PE-Cy5 | BD Biosciences | 555854 |
| FOXP3 PE-Cy5.5 | Invitrogen | 35-4776-42 |
| IL-2 BB700 | BD Biosciences | 566405 |
| CCR5 BV711 | BD Biosciences | 563395 |
| Granzyme B Alx700 | BD Biosciences | 560213 |
| IFN-γ V450 | BD Biosciences | 560371 |
| IL-17a PE-Cy7 | BioLegend | 512315 |
| IL-17F PE-Cy7 | Invitrogen | 25-7169-42 |
| CD56 BV750 | BioLegend | 362556 |
| LAG-3 APC[B] | BioLegend | 369212 |
| TIM-3 APC[B] | BioLegend | 345012 |
| CCR7 (CD197) BV605 | BioLegend | 353224 |
| CD127 BB790 | BD Biosciences | CUSTOM |
| CD69 APC-Fire750 | BioLegend | 310946 |

[A]IL-4 and IL-13 are detected in the same channel.

[B]LAG-3 and TIM-3 are detected in the same channel.

**Supplemental Table 4. Reagents in the 17-color ICS panel.**

| Antibody | Manufacturer | Catalog Number |
|---|---|---|
| AViD[A] | Invitrogen | L34957 |
| CD3 BUV737 | BD Biosciences | 564307 |
| CD4 BUV395 | BD Biosciences | 563550 |
| CD8 BV650 | BD Biosciences | 563821 |
| CD14 BV510[A] | BioLegend | 301842 |
| CD56 BV570 | BioLegend | 318330 |
| CXCR5 PE-Dazzle594 | BioLegend | 356928 |
| PD-1 (CD279) BV605 | BioLegend | 329924 |
| ICOS (CD278) BV711 | BD Biosciences | 563833 |
| CD45RA APC H7 | BD Biosciences | 560674 |
| CCR7 BV785 | BioLegend | 353229 |
| IFN-γ V450 | Becton Dickinson | 560371 |
| TNFα FITC | eBiosciences | 11-7349-82 |
| IL-2 PE | BD Biosciences | 559334 |
| IL-4 PerCP-Cy5.5 | BD Biosciences | 500822 |
| IL-17a PE-Cy7 | BioLegend | 512315 |
| CD154 APC | BD Biosciences | 555702 |
| Granzyme B Alx700 | BD Biosciences | 560213 |

[A]AViD and CD14 are detected in the same channel.

**Supplemental Acknowledgments**

**HVTN 119 Study Group:** The HVTN 119 study team members not listed in the author line includes: HVTN Core, Fred Hutchinson Cancer Research Center: Marnie Elizaga, Laurie Rinn, Jill Zeller, Marianne Hansen, Meg Trahey, Jim Maynard, and Erik Schwab; HVTN Laboratory Program, Fred Hutchinson Cancer Research Center: Nicole Frahm, On Ho; Profectus Biosciences: Susan Sciotto-Brown; Nashville Clinical Research Site: Naomi Prashad; Nashville Community Advisory Board: Ebony Gordon; Boston-Fenway Community Advisory Board: Fred Mazyck; Division of AIDS, NIAID, NIH: Nayri Khairalla and Chris Butler; Statistical Center for HIV/AIDS Research and Prevention, Fred Hutchinson Cancer Research Center: Huguette Redinger, Gina Escamilla, Erika Thommes; and Fenway Clinical Research Site: Laura Michelson.
